# SET-M33 loaded biosynthesized cellulose as effective protection against *S. aureus* biofilm formation

**DOI:** 10.1016/j.bioflm.2026.100351

**Published:** 2026-02-02

**Authors:** Sajad Mohammadi, Alessia Maranesi, Adrianus C.J.M. de Bruijn, Ismael Castañon, Piotr Gierlich, Chiara Falciani, Alessandro Pini, Heleen M.M. van Beusekom, Aldo Ferrari, Wendy W.J. Unger

**Affiliations:** a Department of Pediatrics/Laboratory of Pediatrics, Erasmus MC University Medical Center - Sophia Children’s Hospital, Rotterdam, the Netherlands; b Department of Material Science and Engineering, Universitat Politècnica de Catalunya, Barcelona, 08019, Spain; c Hylomorph AG, Technoparkstrasse 1, 8005, Zurich, Switzerland; d SetLance srl, Siena, 53100, Italy; e Department of Medical Biotechnologies, University of Siena, Siena, 53100, Italy; f Department of Cardiology, Erasmus MC, University Medical Center, Rotterdam, the Netherlands

**Keywords:** Antifouling, Biomaterial, Biopolymer, Implant-associated infection, Localized delivery, Antimicrobial peptides, Antibiotic-resistance

## Abstract

*Staphylococcus aureus* is the most common pathogen responsible for postoperative infections associated with cardiac implantable electronic devices (CIEDs), primarily due to its biofilm-forming capability on implant substrates*.* Protective envelopes, which sustain the local elution of antibiotics, significantly reduce the risk of CIED infection and biofilm formation. However, they are not equipped to counteract emerging bacterial resistance to antibiotics. Antimicrobial peptides (AMPs) can effectively erase contaminating bacteria, without eliciting resistance.

Here, we explored the antimicrobial efficacy of biosynthesized cellulose (BC), a natural biopolymer used in protective envelopes, in combination with two synthetic AMPs: SET-M33D and Mastoparan X (MPX). The BC/AMPs combination inhibited bacterial attachment and subsequent biofilm formation significantly better than native BC or AMP coated titanium substrates, as revealed by full factorial design (FFD) experiments. The outcomes of FFD were used to develop a regression model that estimates the interaction between influential parameters and their impacts on response value. Furthermore, SEM imaging confirmed the superior antibiofilm activity of BC/SET-M33D compared to BC/MPX. We demonstrated that the protective function against *S. aureus* ATCC29213 may be linked to the downregulation of the biofilm associated gene *icaA*.

The results reported demonstrate the feasibility of exploiting BC as AMP carrier for inhibiting biofilm formation in conditions relevant to deployment of CIEDs. While further in vivo evaluation is needed, this approach may offer a promising path to address antimicrobial resistance in the management of post-operative infections associated with CIED implant.

## Highlights

• BC loaded with SET-M33D demonstrated potential effectivity for local delivery of antimicrobial peptide.
• The localized release of antimicrobial peptide from BC can prevent biofilm formation.
• BC/SET-M33D does not induce cytotoxicity and is hemocompatible.

## Graphical abstract

**Figure:**
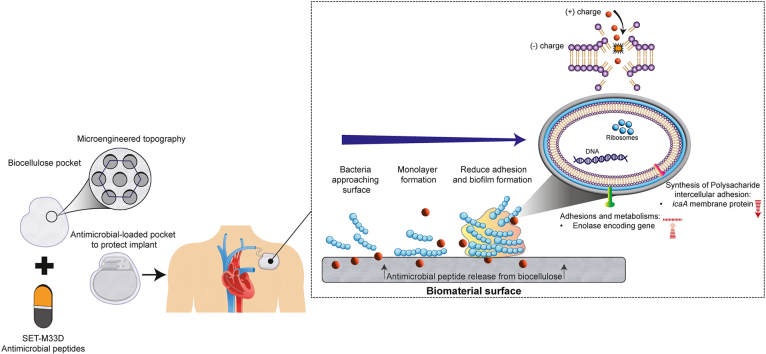

## 1 Introduction

Cardiac implantable electronic devices (CIEDs), such as pacemakers, implantable cardioverter defibrillators, and cardiac resynchronization therapy devices have been used for decades to treat arrhythmias [1]. The outstanding therapeutic benefits of CIED have been tainted by bacterial infections, which result in prolonged hospitalization, implant failure, premature revision, and increased mortality [2,3]. Despite remarkable advances in the pacing technology, device-related infections (DRI) are still considered one of the major difficulties associated with CIED implantation [4]. DRI initiates with opportunistic bacteria entering the device pocket during implant, followed by colonization, biofilm formation, and eventually spreading through the venous system [5]. PCR sequencing identified *Staphylococcus* species as the most common organisms causing DRI [6].

In biofilms, bacteria are shielded by extracellular polymeric substances (EPS), including exopolysaccharides, proteins, extracellular DNA and lipids [7]. This protective mechanism interferes with the host immune system and reduces the efficacy of antimicrobial compounds, potentially leading to bacterial persistence. Consequently, complete removal of the device from the patient's body remains the only treatment option [8], highlighting the importance to prevent colonization and the ensuing biofilm formation.

Current standard of care includes preoperative skin preparation, pocket irrigation with antiseptic solutions and pre- and post-operative antibiotic prophylaxis [[9], [10], [11], [12]]. In high-risk procedures, the use of protective antibacterial envelopes, coated with minocycline and rifampicin (TYRX™, Medtronic), demonstrated superior efficacy against CIED infection [13,14].

Antibiotic-resistant bacteria are posing an existential threat to the use of classic antibiotics in the fight against nosocomial infections, including those that originate at the implant surface [15,16]. In pursuit of alternative solutions to managing infections caused by resistant bacteria, and to replace and/or reduce the use of antibiotics, natural and synthetic antimicrobial peptides (AMPs) have attracted considerable attention in the last decades [[17], [18], [19]].

AMPs are small peptides that carry inhibitory and bactericidal activity against a wide range of pathogens mediated by electrostatic attachment to the bacterial membrane [[20], [21], [22]]. Mastoparan (MP) X is a 14 amino acid peptide (molecular weight of 1556.01 g/mol) extracted from wasp venom active against gram-positive and -negative bacteria, mainly due to increasing bacterial membrane permeability. MPX was found to have potential in inhibiting the colonization of *S. aureus* [23]. On the other hand, the native peptide effect is limited by the proteolytic action of natural proteases such as elastase, secreted by neutrophils and bacteria. Synthetic AMPs such as SET-M33D feature a tetra-branched structure (molecular weight of 5857 g/mol), which strongly improves their resistance to protease activity while preserving antimicrobial activity [24,25].

Biosynthesized cellulose (BC) is a natural polymer featuring nanoscale network structure, high purity, large surface area, robust mechanical properties, and exceptional water retention capacity [[26], [27], [28], [29]]. BC is recognized for its non-inflammatory properties and ability to reduce fibrosis in vivo [30,31]. It provides an effective physical barrier to protect the abiotic surface of the implant from bacterial colonization [32]. However, BC lacks intrinsic antimicrobial properties and can support biofilm formation [33].

The aim of this study was twofold. First, to harness the potential of BC membranes as drug carriers to present AMPs at the CIED interface, specifically targeting *S. aureus* biofilm formation. Second, to specifically evaluate the biocompatibility and hemocompatibility of the AMP delivery system, and to compare the performance of the linear peptide MPX with the tetra-branched peptide SET-M33D in combination with BC membranes.

## 2 Material and methods

### 2.1 Antimicrobial peptides

The SET-M33D peptide, (KKIRVRLSA)_4_K_2_KβA-OH, was synthesized via solid-phase peptide synthesis using a Syro multiple peptide synthesizer (MultiSynTech, Witten, Germany) with standard Fmoc chemistry as previously reported [34,35]. Peptide identity and purity were verified by reversed-phase chromatography on a Phenomenex Jupiter C18 analytical column (300 Å, 5 mm, 25 × 4.6 mm) (KKIRVRLSA)_4_K_2_KβA-NH_2_, RT = 18 min, and confirmed via mass spectrometry using a Bruker Daltonics ultraflex MALDI TOF/TOF (M+ found = 4682.86). Part of the peptide batch was functionalized with fluorescent 5- tetramethyl rhodamine (TAMRA) (Santa Cruz Biotechnology). The dye Fmoc-Lysine conjugated with 5-TAMRA was added to the C-terminal using the standard Fmoc chemistry. The compounds were purified via reverse phase high-performance liquid chromatography (HPLC) using MilliQ 0.01 TFA as eluent A and acetonitrile as eluent B. The SET-M33D and TAMRA- SET-M33D were dissolved in PBS and stored at −20 °C until further use.

TAMRA-labeled and unlabeled MPX AMPs (INWKGIAAMAKKLL) were synthesized and purified by Genscript Inc (New Jersey, USA). The purity was evaluated by HPLC and mass spectroscopy and exceeded 98%. TAMRA-MPX and MPX peptides were dissolved in ultrapure water and dimethyl sulfoxide (DMSO), respectively and stored at −20 °C until further use.

### 2.2 Biocellulose synthesis

BC (Suppl Fig. 1) was produced as previously described [36,37]. Briefly, a micro-structured polydimethylsiloxane (PDMS) mold was placed at the liquid-air interface of the standard static culture of *Acetobacter Xylinum* for 7 days. A highly porous three-dimensional network of randomly coordinated cellulose nanoribbons was formed at the mold surface. Due to the patterns on the mold's surface, BC membrane was featured with micro-wells (2 μm and 20 μm depth and characteristic length, respectively) assembled in a centered hexangular design. After harvesting, the bacterial content of BC membranes was removed by 1 M NaOH at 90 °C for 8 h with subsequent pH adjustment by washing in de-ionized water. Furthermore, the final purification step through washing in aqua ad Iniectabilia (Braun, Germany) was carried out to minimize the endotoxin content of the BC membrane.

### 2.3 Bacterial strains

The focus of this study is on *S. aureus*, the most frequent pathogens causing CIED infections. Therefore, bacterial strains used in current study include *S. aureus* (ATCC29213), MRSA clinical isolates: MW2 [38], Mu50 (rifampicin-resistant) [39] and a MSSA isolate from a patient who received a left ventricular assist device (LVAD) and had been diagnosed with a deep driveline infection [40]. The genetic and phenotypic diversity of the strains are listed in Table 1. The clinical isolates were kindly provided by Prof. W.J.B van Wamel (Dept Medical Microbiology & Infectious Diseases, Erasmus MC, Rotterdam, The Netherlands).

**Table 1:** Strains of *S. aureus* used in the study.

Strain	Genetic background	Description	Reference
ATCC29213	ST5	MSSA, reference strain	
MW2	CC1, USA400	MRSA, clinical isolate	[38]
Mu50	CC5	MRSA and rifampicin-resistant, clinical VISA isolate	[39]
LVAD	ST5	MSSA, clinical isolate	[40]

ST: Sequence Type, CC: Clonal Complex.

### 2.4 Biofilm formation

A stock of *Staphylococcus aureus* (ATCC29213) was thawed under aseptic condition into Lysogeny Broth (LB). To assess the effect of the initial bacterial number on biofilm formation, the stock solution was diluted to the desired concentration in LB, such that subsequently, 1.5 × 10^4^, 1.5 × 10^5^and 1.5 × 10^6^ Colony Forming Units (CFUs)/well in 400 μL were introduced to a 8-well μ-slide (Ibidi GmbH, Germany). Cells were incubated at 37 °C/5% ${\text{CO}}_{2}$ for 8, 24 and 48h to assess the biofilm formation via crystal violet (CV) method, as described before [[41], [42], [43]]. At each timepoint, the planktonic bacteria were removed, and the surfaces were washed twice with 200 μL PBS (pH 7.2) to remove the non-adherent bacteria. Thereafter, the remaining biofilms were fixed with 2% (w/v) sodium acetate for 20 min at RT. After removing the fixative solution, the wells were washed twice with 200 μL PBS and the biofilms were stained with 0.1% CV dye for 15 min at RT. To remove excess dye, wells were rinsed three times with 200 μL PBS. Micrograph images were taken using an inverted microscope (Leica Microsystems, Germany). The CV-stained biofilms were eluted with 200 μL of 95% ethanol for 20 min with subsequent transfer to a flat-bottom 96-well microtiter plate (Greiner Bio-one) and absorbance values were measured at 550 nm using a microplate reader (SpectraMax® iD3, Molecular Devices).

### 2.5 Determination of the minimum biofilm inhibitory concentration of SET-M33D

The minimum biofilm inhibitory concentrations (MBIC) of AMP SET-M33D were determined using a broth microdilution method. Briefly, a two-fold serial dilution of the SET-M33D was performed to achieve a final concentration range from 4 × MIC to 1 × MIC (Suppl Table 1). Each well was inoculated with 200 μL of the bacterial suspension (containing either 1.5 × 10^4^, 1.5 × 10^5^ or 1.5 × 10^6^ CFU) and 200 μL of the peptide solution, resulting in a final volume of 400 μL per well. Controls included LB alone and LB with bacteria. Plates were incubated at 37 °C for 8 and 24 h. After each timepoint, the biofilm formation was measured as mentioned in section 2.3.

### 2.6 Preparation of AMP-treated coupons

The AMP-loaded BC coupons were prepared as previously described with a slight modification [44]. In brief, 8 mm diameter BC membranes that had been stored in H_2_O, were first dried at 80 °C for 20 min, and then placed in 1 mL PBS containing AMPs at concentrations corresponding to 1 × MIC, 2 × MIC and 4 × MIC at RT for 24h. Similarly, 12.7 mm diameter titanium coupons (Biosurface Technologies, USA) were placed in aforementioned SET-M33D and MPX solutions. For both coupons, PBS and PBS containing 1 μM/Rifampicin (Rifadin, Sanofi B.V) were used as control groups.

### 2.7 Design of experiment and antibiofilm activity of AMP-treated BC and Ti

A full factorial design (FFD) was used to identify the influence of biomaterial type, AMP type and their concentration — to estimate the interaction between them. An FFD can be used to determine the effect of each factor on the response, as well as its variation as a result of changes in the levels of other factors [45]. The levels are usually referred to as either low and high, or −1 and +1. In this scenario, the FFD is said to be symmetric, meaning that all the factors studied have the same number of levels. In contrast, the design is asymmetrical when each factor is assigned a different number of levels [46]. In the current work, we assumed the biomaterial surface, antimicrobial peptide and concentration of the peptide as the influential factors on biofilm inhibition. The detailed variables and their levels for the experiment are reported in Table 2. After defining the independent variables, their levels and setting two replicates for each, a total number of 24 experimental points (also known as runs) with all possible combinations were proposed in randomized order by Design-Expert® software (Suppl Table 2).

**Table 2:** Experimental factors and their levels used in Full Factorial Design.

Variables	Coded symbol	Levels		
		Low (−1)	Medium (0)	High (+1)
Biomaterial	A	BC		Ti
AMP type	B	M33-D		MPX
AMP concentration ( × MIC)	C	1	2	4

Following experimental design, sterile BC and titanium coupons were treated with AMPs, as explained in section 2.5. Coupons were placed in a 24-well plate, inoculated with 1.5 × 10^5^ CFU of *S. aureus* and incubated for 6–8 h at 37 °C to allow the biofilm to establish. After removal of the planktonic bacteria 1 mL of fresh LB was added to each well, followed by 16–18 h incubation to mature the biofilm. This approach was applied because the biofilm development process can be divided into several stages: attachment and adhesion (0–6h), aggregation and bacterial proliferation (6–16h), biofilm maturation and structuring (16–24h) [47,48]. Coupons treated with PBS and 1 μM rifampicin served as control. After 24 h incubation, the medium was removed and the coupons were rinsed twice with 1 mL PBS each time. The coupons were then placed in 1 mL PBS, vortexed for 30 s, sonicated (Branson 5210, USA) for 2 min at 40 kHz and vortexed again for 30 s to disaggregate the biofilm. Subsequently, the sonicated fluids were serially diluted in PBS and 20 μL of the dilutions were plated on LB agar plates and incubated for a further 24 h at 37 °C/5% ${\text{CO}}_{2}$. After incubation, the number of CFU/Area(cm^2^) was calculated and reported as the response for further FFD analysis. The experimental data were fitted to the following first-order polynomial equation: $\sqrt{Y}=\beta_{0}+\beta_{1}A+\beta_{2}B+\beta_{3}C+\beta_{12}\text{AB}+\beta_{13}\text{AC}+\beta_{23}\text{BC}+\beta_{123}\text{ABC}$

Here, Y represents the response value, $\beta_{0}$ is the intercept, $\beta_{1},\beta_{2}$ and $\beta_{3}$ are the coefficients for the influential factors. $\beta_{12}$*,* $\beta_{13}$*,* $\beta_{23}$ *and* $\beta_{123}$ signify the coefficients for two-way and three-way interactions among the factors, respectively.

### 2.8 Antibiofilm activity of SET-M33D-loaded BC against clinical isolates

The ability of SET-M33D-treated BC to inhibit the formation of biofilm by MW2, Mu50 and LVAD clinical isolates was evaluated as described in section 2.7. Comparative analysis was performed against Ti/SET-M33D. The efficacy of SET-M33-loaded BC/Ti in inhibiting bacterial adhesion and biofilm formation was determined by a reduction in both CFU and bacterial metabolic activity.

BC and Ti were treated with 2 × and 4 × MIC of SET-M33D, 4 × MIC of rifampicin (for MW2 and LVAD isolates) and 19.5 μM rifampicin (for the rifampicin-resistant strain Mu50). PBS-treated coupons were used as control. In addition to determining the CFU/area, the metabolic activity of the bacteria within the dislodged biofilm on the coupons’ surfaces was assessed. To this end, 100 μL of sonicated fluid (dislodged biofilm) was transferred to a 96-well flat-bottomed plate. Then, 100 μL of resazurin at a concentration of 3 μg/mL was added to each well. The plate was incubated at 37 °C/5% CO_2_ until the color of the control wells turned pink. Fluorescent intensity (FI) was then measured using a microplate reader (SpectraMax® iD3, Molecular Devices) at an excitation wavelength of 530 nm and an emission wavelength of 590 nm. Metabolic activity of bacteria was calculated using the following equation:

$$Metabolic\;activity\;\%=\frac{{\text{FI}}_{\text{sample}}-{\text{FI}}_{\text{negatice}\;\text{control}}}{{\text{FI}}_{\text{positive}\;\text{cntrol}}-{\text{FI}}_{\text{negatice}\;\text{control}}}\times 100$$

### 2.9 AMP loading and release efficiency

To evaluate the amount of peptide loaded onto BC, TAMRA-labeled SET-M33D and MPX were used. BC disks (n = 3) were cut in 8 mm diameter and submerged in 1 mL AMP solution at concentrations corresponding to 4 × MIC for SET-M33D and MPX. The samples were then incubated at RT, and at certain time points, aliquots of the solution were taken, and the absorbance was measured at 550 nm wavelength using a spectrophotometer. The amount of AMP in the solution was estimated from the calibration curves (from 0 to 1000 μg/mL) prepared for each peptide. The amount of peptide loaded on the BC was determined using the following equation:

$$\text{Loaded}\;\text{peptide}\;\%=1-\frac{C_{t}}{C_{0}}\times 100$$

Where $C_{t}$ represents concentration at specific time point and $C_{0}$ shows initial concentration.

Subsequently, the AMP release from BC was investigated, as previously described [49]. The AMP-loaded disks were placed in 1 mL sterile PBS and incubated at 37 °C. Aliquots were taken at predetermined time points and the amount of AMP in the solution was calculated according to the calibration curve and below equation:

$$\text{Released}\;\text{peptide}\;\%=\frac{C_{t}}{C_{0}}\times 100$$

Where $C_{t}$ represents concentration at specific time point and $C_{0}$ shows the concentration of AMP loaded into BC disks. Thereafter, the cumulative drug release data were calculated and fitted to various release kinetic models including zero order, first order, Higuchi and Korsmeyer–Peppas (KP) [50], using libraries such as NumPy for numerical computations [51] and SciPy for optimization [52] in Python V3.10. The corresponding equations for the mathematical models are as follows:

$$\text{Zero}-\text{order}:Q_{t}={Q_{0}+K}_{0}t$$

$$\text{First}-\text{order}:Q_{t}=Q_{0}e^{-k_{1}t}$$

$$\text{Higuchi}:Q=k_{H}t^{1/2}$$

$$\text{Korsmeyer}-\text{Peppas}:\frac{M_{t}}{M_{\infty }}=k_{KP}t^{n}$$

Where Q_0_ is the initial amount of drug, Q_t_ is the amount of drug at time t, k is released constant, t is time, $\frac{M_{t}}{M_{\infty }}$ is drug release fraction at time t and n is release exponent.

### 2.10 Scanning electron microscopy (SEM)

Bacterial adhesion (*S. aureus* ATCC29213) on BC was imaged with SEM. In brief, BC was treated with AMPs, and bacterial biofilm was grown onto their surface as described above. BC treated with rifampicin or without antimicrobial compound was used as control. After 24h incubation, coupons were rinsed with PBS and the biofilm was fixed in 1 mL 2% glutaraldehyde and 4% paraformaldehyde in a 24-well plate for 24h at 4 °C. Thereafter, samples were rinsed with 0.1 M sodium cacodylate trihydrate (pH 7.2; Sigma-Aldrich) with subsequent dehydration in increasing concentrations of ethanol (1 × 10%, 30%, 50%, 70% and 90% for 20 min and 2 × 100% for 30 and 60 min). The ethanol was then rinsed off and the samples were incubated in 1 mL hexamethyldisilazane (HMDS) (Sigma-Aldrich) for 10 min. After 10 min, HMDS was replaced with 1 mL fresh HMDS and the plate was incubated without lid at RT until the samples were completely dried. The samples were then mounted onto aluminum stubs using double-sided carbon adhesive and sputter-coated with gold to make them conductive. SEM imaging was performed using a JSM-6060LV microscope (JEOL; Japan) under high vacuum conditions and with an accelerating voltage of 5 kV.

### 2.11 RT-PCR

*S. aureus* biofilm formation is a complex process that is mediated by various regulatory mechanisms, such as microbial surface components recognizing adhesive matrix molecules (MSCRAMMs) and polysaccharide intercellular adhesion (PIA). While *Eno* belongs to MSCRAMM family and encodes a surface-associated protein that is essential for bacterial attachment, *icaA* regulates *S. aureus* biofilm formation through encoding proteins involved in PIA production [53,54]. To assess the effect of treating BC and Ti with AMPs on the expression of biofilm-associated genes, biofilm was formed on the aforementioned biomaterials as described in section 2.6, using *S. aureus* ATCC29213. After incubation, coupons were washed and placed in 1 mL PBS for sequential vortex and sonication. The coupons were then removed, and the sonicated fluid was centrifuged at 5000×*g* for 10 min. Total RNA was extracted from the bacterial cells using the NucleoSpin® RNA kit (MACHEREY-NAGEL, Germany) according to the manufacturer's instructions. The concentration and purity of the RNA was then checked using a quantification spectrophotometer (DeNovix®, USA). The SensiFAST™ cDNA synthesis kit (meridian BIOSCIENCE) was used to synthesize cDNA from 200 ng of total RNA. qPCR reactions were performed using 2.5 μL cDNA, 12.5 μL SYBR green, 0.5 μL MgCl_2_ (SensiMix™), and 0.75 μL of forward and reverse primers (Table 3) at a concentration of 10 μM in clear hardshell PCR plates (Bio-Rad Inc, USA) in a CFX96 Real-Time system C1000 (Bio-Rad). cDNA of *S. aureus* detached from untreated material was used as control. The following conditions were applied: 10 min at 95 °C, followed by 40 cycles of 15 s at 95 °C and 1 min at 60 °C. Amplicon and melting curves were inspected visually, and technical replicates were considered adequate if they differed no more than 1 cycle. The quantification cycle (Cq) was determined using the Bio-Rad CFX manager algorithm. The data were normalized to 16S rRNA as reference gene, and expressed as fold change versus control biofilm cultures growing on untreated BC/Ti.

**Table 3:** Primer sequences.

Gene	Forward primer	Reverse primer	Amplicon size (bp)
16s rRNA	CAAAACTACTGAGCTAGAGTACG	GCCACTGGTGTTCCTTCCTA	89
*icaA*	ACACTTGCTGGCGCAGTCAA	TCTGGAACCAACATCCAACA	188
*Eno*	ACGGTGCAAAACGTACAG	GTTTCCAACCATCCCAGTC	117

### 2.12 Biocompatibility

The biocompatibility of SET-M33D-treated BC and Ti was investigated by measuring the metabolic activity of the THP-1 human monocytic cell line and primary dermal fibroblasts, derived from a 63-year old female donor. BC and Ti disks were treated either with 15 μM AMP or PBS and incubated in a 24-well plate O/N at 37 °C/5% CO_2_. The samples were then washed with 1 mL PBS and 5 × 10^5^ THP-1 cells were seeded directly onto the biomaterial surface. Whereas, the samples were added to the seeded fibroblasts (3500 cell/cm^2^) in IMDM, supplemented with 10% FCS. The plates were incubated at 37 °C with 5% CO_2_ for 24 h. After incubation, the THP-1 cells were thoroughly resuspended and 100 μL were transferred to a 96-well flat-bottomed plate. Subsequently, 100 μL of resazurin at a concentration of 1 μg/mL was added to each well. For fibroblasts, the samples were removed from wells and 1 μg/mL resazurin was added to the cells. The plates were incubated at 37 °C/5% CO_2_ until the color of the control wells turned pink. The FI was measured at an excitation wavelength of 530 nm and an emission wavelength of 590 nm using a microplate reader (SpectraMax® iD3, Molecular Devices). The metabolic activity of cells was calculated by the following equation:

$$Metabolic\;activity\;\%=\frac{{\text{FI}}_{\text{sample}}-{\text{FI}}_{\text{negatice}\;\text{control}}}{{\text{FI}}_{\text{positive}\;\text{cntrol}}-{\text{FI}}_{\text{negatice}\;\text{control}}}\times 100$$

### 2.13 Hemocompatibility

The hemolytic potential of the SET-M33D-loaded BC was measured by a direct in vitro hemolysis assay, according to the previous report and ISO 10993-4 [55]. Briefly, 5 mL of human whole blood was used for this assay. Red blood cells (RBCs) were isolated from whole blood by centrifuging at 1000×*g* for 10 min for three repetitions. The supernatant was discarded each time, and the pellet was washed with calcium- and magnesium-free PBS. The RBCs obtained were then 10-fold diluted in PBS to a final volume of 10 mL for further use. BC samples (n = 3) were loaded with SET-M33D at concentrations corresponding to 2 × MIC and 4 × MIC as described above. Samples treated with PBS served as untreated controls. BC discs were then placed in a 24-well plate and conditioned with 800 μL PBS followed by the addition of 200 μL RBCs. RBCs dispersed in 800 μL PBS and deionized water served as negative and positive control, respectively. The plate was then incubated at 37 °C for 2 h. All suspensions were then transferred to 1.5 mL Eppendorf tubes and centrifuged at 1000×*g* for 5 min. 200 μL of the supernatant was then transferred to a flat-bottomed 96-well plate and the absorbance of the released hemoglobin in the supernatant was measured at 540 nm by a microplate reader (SpectraMax® iD3, Molecular Devices). The % hemolysis was determined using the following equation:

$$\text{Hemolysis}\;\text{ratio}\;\%=\frac{{\text{Sample}}_{\text{abs}}-{\text{Negative}\;\text{control}}_{\text{abs}}}{{\text{Positive}\;\text{control}}_{\text{abs}}-{\text{Negative}}_{\text{abs}}}\times 100$$

### 2.14 Statistical analysis

The results of the full factorial design of experiment were analyzed in Design-Expert® software, where analysis of variance (ANOVA) and linear regression model with square root transformation were employed to determine the significance of factors and their interactions. GraphPad Prism (V9.0.0, GraphPad Software Inc, USA) was used for all other statistical analyses. Comparisons were conducted using one-way or two-way ANOVA. Either Sidak or Tukey correction was carried out when multiple comparison tests were performed. A *p*-value <0.05 was considered as statistically significant.

## 3 Results

### 3.1 Effects of initial bacterial load on of *S. aureus* biofilm formation dynamics and antimicrobial susceptibility

**Fig. 1:**
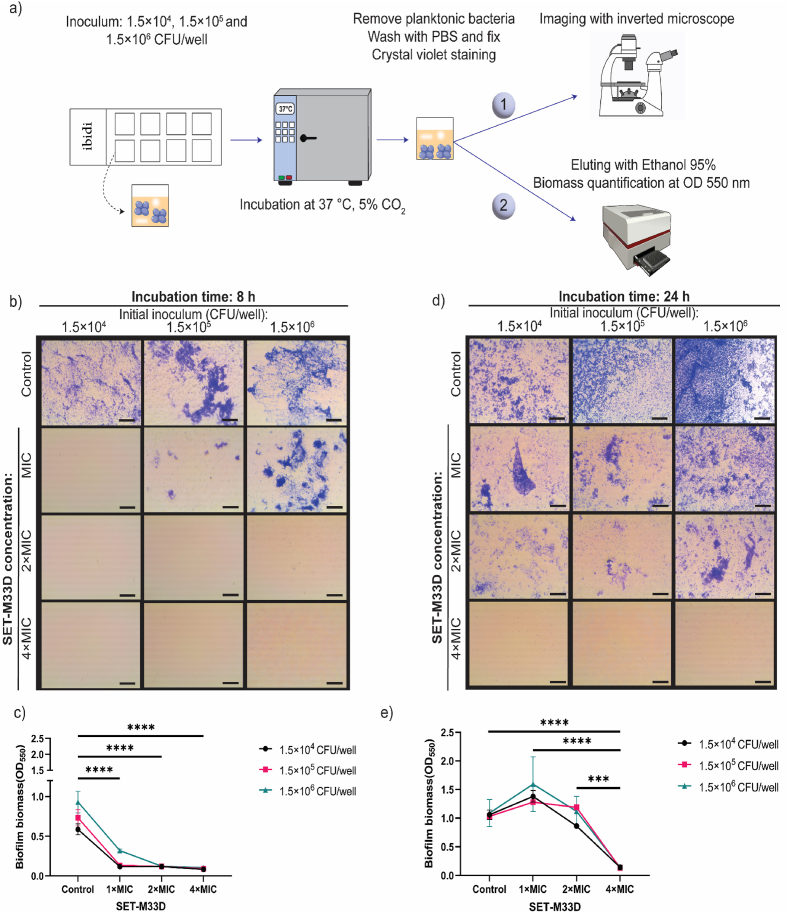
**The effect of initial load of bacteria on the dynamics of *S. aureus* biofilm formation, indicating its importance in starting infection and antimicrobial susceptibility.** (a) Schematic representation of the experiment. (b and d) representative microscopic images of established biofilms stained with crystal violet, and treated with indicated dose of SET-M33D after 8h and 24h. Scale bar 500 μm. (c and e) quantification of biofilm biomass at 8h and 24h via measuring the OD550 of eluents. Graphs show means ± SEM of two independent experiments, each performed with technical replicates. (∗∗∗) p < 0.001 and (∗∗∗∗) p < 0.0001. (For interpretation of the references to color in this figure legend, the reader is referred to the Web version of this article.)

The kinetics of the biofilm formation and its structural development are influenced by the initial amount of bacteria landing on a substrate [56,57]. To evaluate the effect of the initial microbial load and the growth dynamics of *S. aureus* biofilm formation, tissue culture plastic was first used in the absence of the confounding variables introduced by a more complex biomaterial surface (Fig. 1). This also offered the required optical transparency for the microscopic inspection of the biofilm.

Three different bacterial inocula, providing a step-wise increase by one logarithmic unit (i.e. 1.5 × 10^4^, 1.5 × 10^5^, and 1.5 × 10^6^ colony-forming units (CFU)) were evaluated. The resulting biofilm biomass was quantified after incubation periods of 8, 24, and 48 h, rendering a matrix of three conditions and three subsequent time points.

Crystal Violet staining and microscopic observation showed that, independently from the initial bacterial load, a biofilm was present at 8h post-incubation (Suppl Fig. 2a). At this point in time, the biofilm structure appeared similar for all different initial CFU, with weaker and filamentous structures prominent in cultures started with 1.5 × 10^4^ CFU. Quantification of biofilm biomass confirmed the visual observation: cultures started with 1.5 × 10^4^ CFU *S. aureus* displayed lower biomass than those started with 1.5 × 10^5^ and 1.5 × 10^6^ CFU (Suppl Fig. 2b). The biofilm forming capacity of *S. aureus* increased over time and, at 24h, the established biofilms showed a denser structure, with highest biomass measured when cultures were started with 1.5 × 10^6^ CFU (*p* < 0.01). At 48h the differences in biofilm biomass between the cultures levelled up (Suppl Fig. 2b).

We next investigated whether the initial bacterial load had an impact on the susceptibility to AMP antimicrobial treatment. Since a significant increase in biofilm mass was observed within the first 24h of culture, the effects of AMP treatment were therefore assessed within this time frame. *S. aureus* bacterial loads of 1.5 × 10^4^, 1.5 × 10^5^ and 1.5 × 10^6^ CFU/well were treated with the tetra-branched AMP SET-M33D at concentrations equal to 1 × , 2 × and 4 × the minimum inhibitory concentration (MIC) values (Suppl Table 1). Crystal violet staining of the biofilm revealed a dose-dependent reduction in biofilm biomass, with considerable differences stemming from incubation time and initial bacteria load. Treatment with the SET-M33D AMP, especially at 2 × MIC and 4 × MIC, appeared sufficient to inhibit biofilm formation within 8h, irrespective of the initial load (Fig. 1b and c). Even at 1 × MIC, inhibition of biofilm formation was detected, with strongest effect in wells containing 1.5 × 10^4^ CFU. However, examination at 24h indicated that treatment with SET-M33D at 1 × or 2 × MIC had been ineffective in killing all bacteria, as equal biofilm biomass was detected as in untreated control wells (Fig. 1c and d). Only at 4 × MIC, crystal violet intensity was still significantly decreased at 24h (*p* < 0.0001), indicating sustained near-complete biofilm inhibition at this concentration.

### 3.2 AMP-treated BC demonstrated antibiofilm activity

**Fig. 2:**
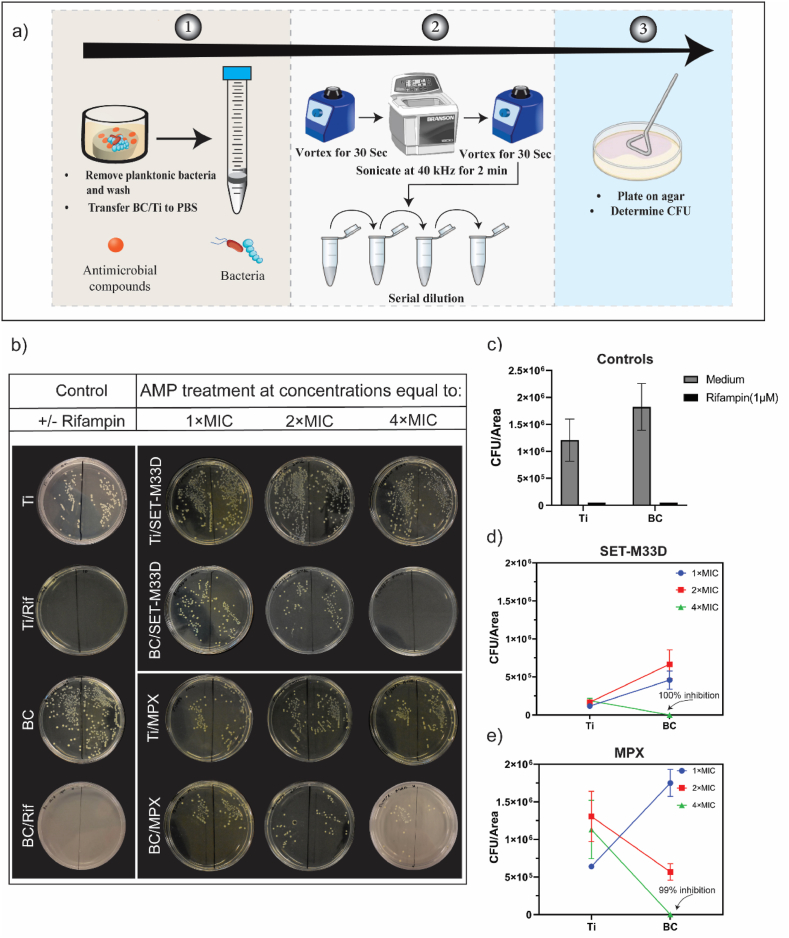
**Bacterial attachment and biofilm formation on AMP treated BC and Ti.** a) Schematic of experiment to dislodge formed biofilm on the biomaterial surface and preparation for plating. b) CFU counting on agar plate as indicators for biofilm formation on BC or Ti, treated with the indicated dose of SET-M33D and MPX antimicrobial peptides. c) control conditions where BC and Ti were either treated with 1 μM rifampicin or PBS. Each agar plate shows technical replicates. Interaction plots showing the comparison between the performance of Ti and BC at different concentration of d) SET-M33D and e) MPX. Graphs show mean CFU/area ±SEM of two independent experiments. Inhibition % of AMP-loaded BC at 4 × MIC was compared to unloaded BC and indicated on the plots.

To evaluate the effective configuration of BC/AMPs in inhibiting bacterial attachment and biofilm formation, BC (representing the porous protective envelope) was treated with SET-M33D at concentrations equivalent to 1 × MIC, 2 × MIC and 4 × MIC. Although SET-M33D at 1 × and 2x MIC were ineffective in inhibiting biofilm formation when applied as mono-therapy, it is conceivable that it may act synergistically with BC at this dose. To evaluate potential hindrance due to the size/structure of the tetra-branched peptide SET-M33D in this setting, we compared its performance with the smaller, linear peptide MPX. These data were then compared with those from Ti (representing medical devices) treated under the same conditions. As crystal violet staining is not applicable in this configuration, the antibiofilm activity of the AMP-treated biomaterials was instead evaluated through a quantitative analysis of bacterial colonies cultivated on agar plates. This analysis was performed following the detachment of viable adherent bacteria from the biofilms recovered from BC and Ti surfaces (Fig. 2a and b). In the absence of an antimicrobial compound, bacterial adhesion and the formation of a biofilm was observed on the surfaces of both BC and Ti (Fig. 2b and c). Albeit not statistically significant, a reduced level of *S. aureus* adhesion was observed on Ti compared to BC. The administration of the antibiotic rifampicin, as control, to both BC and Ti resulted in the complete inhibition of bacterial adhesion and biofilm formation (Fig. 2b + c). The performance of AMPs in eliminating bacterial adhesion and biofilm formation on Ti and BC is illustrated in the form of interaction plots (Fig. 2d and e). SET-M33D demonstrated superior performance in comparison to MPX on both BC and Ti surfaces. While *S. aureus* was able to form biofilms onto BC/SET-M33D at 1 × MIC and 2 × MIC, the loading of BC with SET-M33D at 4 × MIC resulted in complete inhibition (100%) of bacterial adhesion and biofilm formation (Fig. 2b + d). Although bacterial counts were ≈ 4-fold lower when SET-M33D was used for treatment of Ti surfaces compared to BC, on the Ti surfaces, SET-M33D did not achieve complete inhibition of bacterial adhesion and biofilm formation (Fig. 2d and e). BC/MPX demonstrated dose-dependent effects, evidenced by its ability to reduce viable bacteria (Fig. 2e). However, its efficacy in preventing bacterial outgrowth on Ti was significantly inferior to that of SET-M33D, as shown in Fig. 2d and e.

**Fig. 3:**
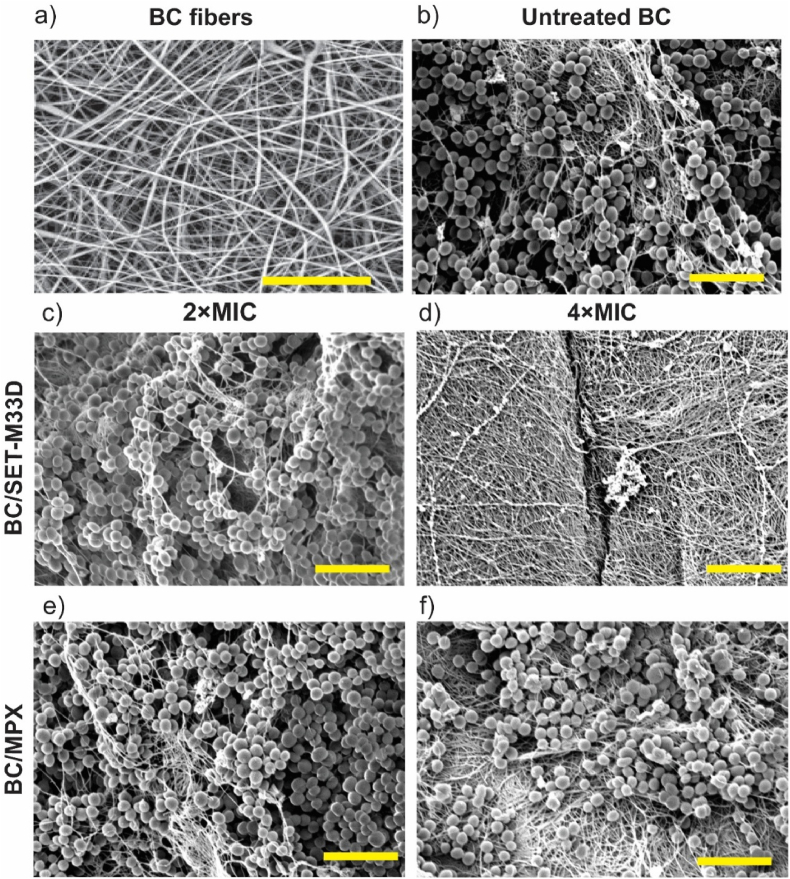
**Visualization of bacterial adhesion (*S. aureus*** ATCC29213**) on unloaded and AMP-loaded BC with** SEM analysis. a) Plain BC showing its fibrous structure. Scale bar: 20 μm. b) Untreated BC and bacterial attachment. c) *S. aureus* attachment on BC treated with 2 × MIC SET-M33D. d) Inhibition of bacterial attachment on BC when treated with 4 × MIC SET-M33D. e) and f) bacteria adhesion on BC after treatment with 2 × and 4 × MIC MPX, respectively. Scale bar in b–f: 5 μm.

To verify the impact of SET-M33D and MPX peptides on alterations in bacterial adhesion and biofilm formation on porous and fibrous BC (Fig. 3a), scanning electron microscopy (SEM) was conducted. As shown in Fig. 3b, SEM imaging confirmed the presence of *S. aureus* clusters on untreated BC. A similar pattern was observed in BC samples treated with 2 × MIC of either peptide (Fig. 3c and e), where bacterial colonization and structural integrity were maintained, indicating that this concentration of AMPs was insufficient to disrupt bacterial adhesion or biofilm integrity. These findings align with CFU counts obtained after biofilm detachment and agar plating (Fig. 2d and e). In contrast, complete inhibition of bacterial attachment was observed on the BC surface treated with 4 × MIC of SET-M33D (Fig. 3d), exhibiting similar antibiofilm activity as observed for rifampicin-loaded BC (Suppl Fig. 3). For MPX at 4 × MIC, SEM revealed a reduced bacterial load and partial inhibition of biofilm formation (Fig. 3f), despite CFU analysis (Fig. 2e) indicating a near-complete reduction in viable cells.

### 3.3 Full factorial design to estimate effects of biomaterial type, AMP type, AMP concentration and interactions on biofilm formation

Bacterial attachment and biofilm formation on implantable materials are influenced by multiple factors, including physico-chemical properties, environmental conditions and surface morphometry [58]. Here, a full factorial design was employed to evaluate the effects of the biomaterial type, AMP type, AMP concentration, and their interactions on biofilm formation. The dependent variable – biofilm formation - was significantly affected by the selected independent variables. Based on the enumeration of viable bacteria from the biomaterial surfaces, the response polynomial coefficients were calculated. A detailed analysis of variance (ANOVA) is presented in Table 4. The model F-value of 22.96 indicates that the regression model is statistically significant, with only a 0.01% probability that such a high F-value could occur due to random noise. Model terms with *p*-value <0.05 were considered significant contributors to the responses. In this case, AMP type, AMP concentration, the interactions between biomaterial and AMP type, biomaterial and AMP concentration, as well as the three-way interaction, were all significant.

**Table 4:** Full factorial ANOVA.

Source	Sum of Squares	df	Mean Square	F-value	p-value
**Model**	3.843 × 10^6^	11	3.493 × 10^5^	22.96	**< 0.0001**
A-Biomaterial type	57919.17	1	57919.17	3.81	0.0748
B-AMP type	9.688 × 10^5^	1	9.688 × 10^5^	63.67	**< 0.0001**
C-AMP Concentration	8.728 × 10^5^	2	4.364 × 10^5^	28.68	**< 0.0001**
AB	2.360 × 10^5^	1	2.360 × 10^5^	15.51	**0.0020**
AC	1.370 × 10^6^	2	6.848 × 10^5^	45.01	**< 0.0001**
BC	70185.24	2	35092.62	2.31	0.1420
ABC	2.674 × 10^5^	2	1.337 × 10^5^	8.79	**0.0045**
**Pure Error**	1.826 × 10^5^	12	15215.44		
**Cor Total**	4.025 × 10^6^	23			

The fit statistics (Suppl Table 3) further confirmed the adequacy of the linear regression model. The R^2^ = 0.9546 indicates that approximately 95% of the variation in the dependent variable is explained by the predictors. The predicted R^2^ (0.8186) is in reasonable agreement with the adjusted R^2^ (0.9131), with a difference of less than 0.2, indicating good model predictability [59]. The signal-to-noise ratio, calculated to be 15.147, exceeds the threshold of 4, suggesting that the model provides a reliable estimate of the response. These findings are further supported by the normal probability plot (Suppl Figure 4a), which shows that the residuals follow a normal distribution along the 45° regression line, while the plot of residuals versus run order (Suppl Figure 4b), displays a random scatter within defined boundaries, indicating the absence of lurking variables that might compromised the responses and model accuracy [60].

To identify the relative effect of factors, the relation between the dependent variable and the independent parameters are expressed in the following polynomial equation, in terms of coded factors:

$$\sqrt{Y}=+644.1-49.13\times A+200.92\times B+139.21\times C_{1}+130.44\times C_{2}-99.16\times AB+262.06\times {AC}_{1}+53.62\times {AC}_{2}+76.34\times {BC}_{1}-34.18\times {BC}_{2}+146.79\times {ABC}_{1}-96.83\times {ABC}_{2}$$

Despite the *p*-value being greater than 0.05 for biomaterial in this specific study, this term was not excluded from the regression model due to its significant contribution to the response variable through interaction with other parameters and its central role in the research question. Similarly, the BC (AMP type – Amp Concentration) term was retained in the model, because maintaining hierarchical consistency is necessary to accurately approximate the three-way interaction (term ABC). The coefficients indicate the magnitude and direction of the influence of each factor and its interactions on the response variable.

### 3.4 Antibiofilm activity of BC/SET-M33D against clinical isolates

**Fig. 4:**
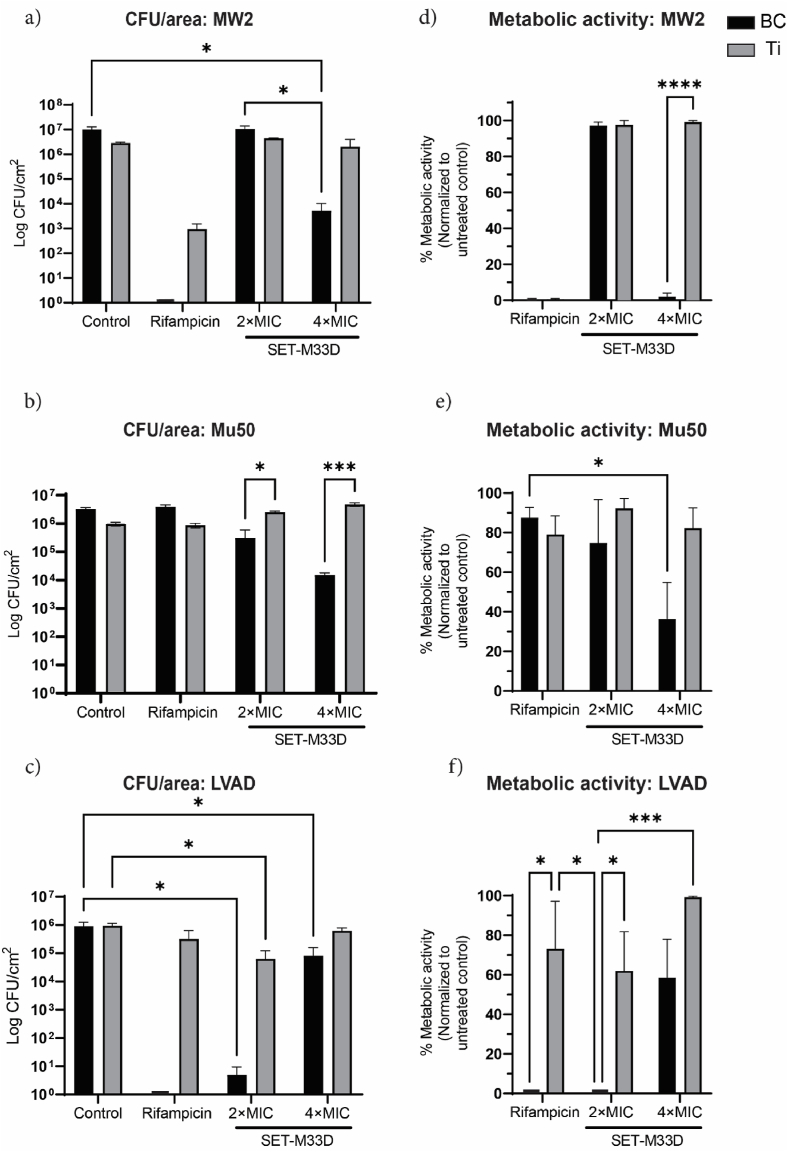
**Antibiofilm activity of AMP treated BC against MRSA and MSSA clinical isolates.** CFU counting on agar plate as indicators for biofilm formation on BC or Ti, treated with the indicated dose of SET-M33D antimicrobial peptides for a) the MRSA isolate MW2, 3.84 ± 0.86 log_10_ reduction in biofilm formation, compared to untreated control. b) Mu50, 2.33 ± 0.08 log_10_ reduction in bacterial adhesion and biofilm formation compared to the rifampicin-loaded samples. c) LVAD, full biofilm inhibition on the rifampicin-loaded BC and a 5.38 ± 0.57 log_10_ reduction in the CFUs of the dislodged biofilm from the BC surface when loaded with a 2 × MIC SET-M33D and infected with *S. aureus* isolated from a patient who received an LVAD and was diagnosed with a deep driveline infection. d) Metabolic activity of MW2 as a result of loading/treating BC and Ti with SET-M33, indicating a ≈98% reduction. e) Metabolic activity of the rifampicin-resistant MRSA isolate, Mu50. BC/SET-M33 at 4 × MIC led to 64 ± 19% reduction in the metabolic activity of Mu50. f) Metabolic activity of the LVAD-isolated *S. aureus*, demonstrating a significant reduction in the case of rifampicin- and AMP-loaded BC. Graphs show the means ± SEM of two independent experiments, each of which was performed in triplicate. (∗) p < 0.05, (∗∗∗) p < 0.001 and (∗∗∗∗) p < 0.0001.

Next, we investigated the ability of BC/SET-M33D to inhibit biofilm formation by both MRSA and MSSA clinical. Specifically, the MRSA strains isolates MW2 and Mu50 (rifampicin-resistant) and the MSSA LVAD isolate were tested in accordance with the FFD outcomes. While BC/SET-M33 at 4 × MIC achieved complete biofilm inhibition against reference strain *S. aureus* ATCC29213, this level of inhibition was not replicated when clinical isolates were examined. Determining the bacterial count of the detached biofilm from the surfaces of the samples showed that this combination was ineffective at 2 × MIC of AMP for both Mu50 and MW2. However, BC/AMP at 4 × MIC outperformed the untreated BC and Ti/AMP. The CFU/area of MW2 detached from BC/SET-M33 at 4 × MIC was 3.84 ± 0.86 log_10_ lower than from the untreated control, indicating a log reduction in viable bacteria that was approximately seven times higher than that achieved with Ti/AMP at 4 × MIC (Fig. 4a). The metabolic activity assay results were in line with the CFU determinations, showing 2 ± 1.6% and 99 ± 1.8% activity for BC/AMP and Ti/AMP at 4 × MIC, respectively (Fig. 4d).

Conversely, Mu50 has been shown to be resistant to rifampicin [61]; highlighting the limitation of antibiotics in preventing biofilm formation and infection in patients receiving implants. A 2.33 ± 0.08 log_10_ reduction in CFU of detached biofilm was observed at 4 × MIC for BC, whereas no activity was detected in rifampicin-treated BC or Ti (Fig. 4b). Furthermore, assessment of metabolic activity demonstrated the same trend: 36 ± 19% and 96 ± 4.2% for BC/AMP and BC/Rif, respectively (Fig. 4e).

Although rifampicin has been used to control in vivo infections caused by *S. aureus*, treating Ti with rifampicin showed minimal inhibition of biofilm formation against the LVAD-isolated strain (Fig. 4c and f). Conversely, loading BC with an identical concentration of rifampicin resulted in full biofilm clearance. A similar trend was observed when the antibiofilm activity of AMP-treated BC and Ti was evaluated. Lower biofilm formation was consistently detected on BC/SET-M33D at 2 × MIC (5.38 ± 0.57 log_10_ reduction), followed by a 99.9 ± 0.14% reduction in the metabolic activity of the bacteria. Nevertheless, the metabolic activity of the bacteria dislodged from biofilms formed on Ti surfaces treated with 2 × MIC of SET-M33D decreased by only ≈38%, on average.

Overall, these results are significant in three ways. Firstly, they validate the FFD model developed to systematically estimate the interaction between AMP and biomaterials in order to predict antimicrobial performance. Secondly, they confirm the importance of the physical structure of the biomaterial in governing the antimicrobial properties; as demonstrated by BC's superior activity to prevent biofilm formation when combined with antimicrobial agents. Thirdly, they demonstrate that the proposed combination (BC/SET-M33D) can inhibit biofilm formation by MRSA and MSSA clinical isolates, which are real-world pathogens and relevant to clinical practice.

### 3.5 In vitro AMP loading into BC, release kinetics and mathematical modeling of release mechanisms

The obtained results in section 3.2 demonstrated a secure attachment and biofilm formation onto BC/AMPs, particularly at lower concentrations of AMPs. This phenomenon likely occurred due to incomplete release and insufficient availability of the AMP to demonstrate either bactericidal or anti-adhesion activity. The efficacy of AMPs in preventing bacteria adhesion and subsequent biofilm development is highly dependent on the availability of sufficient molecules to interact with the target bacteria. Consequently, if AMP loading is insufficient or if release from the BC is incomplete, the concentration required to exert bactericidal activity or inhibit adhesion may not be achieved.

**Fig. 5:**
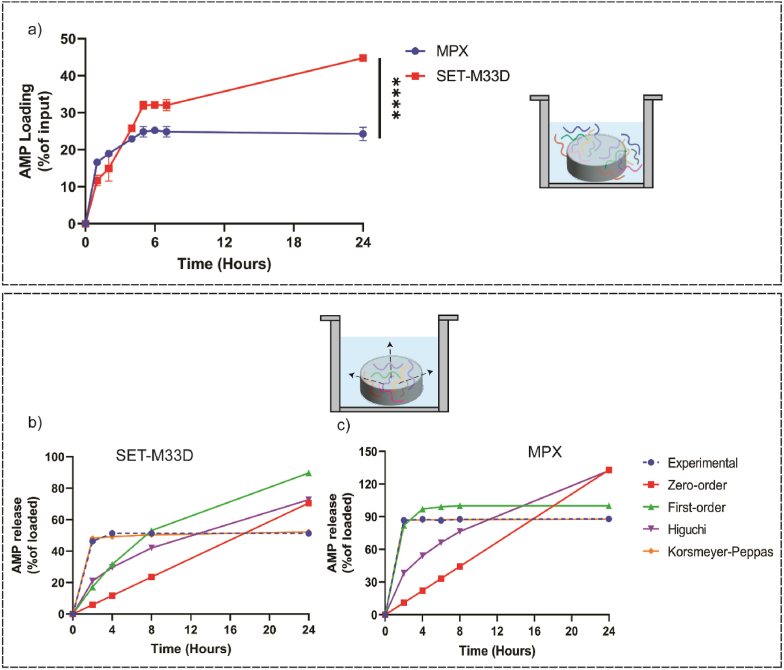
**In vitro assessment of the efficiency of AMP loading into BC and mathematical modeling of release kinetics.** a) Loading antimicrobial peptides into BC coupons using an immersion method and their loading efficiency. In vitro AMP release data for b) SET-M33D and c) MPX, and fitted to Zero-order, First order, Higuchi and Korsmeyer-Peppas mathematical models. Mean of triplicates ± SEM, (∗∗∗∗) *p* < 0.0001.

To address this, the total amounts of SET-M33D and MPX loaded in BC were quantified and expressed as a percentage of the initial input (Fig. 5a). After 24h, the loading efficiency of MPX was approximately 25%, while SET-M33D a higher loading efficiency of approximately 45%. Subsequent in vitro release studies were conducted in PBS at 37 °C, and results were shown as percentage of AMP released relative to the amount initially loaded (Fig. 5b and c). MPX displayed a rapid release profile, with approximately 90% released within 2 h, indicative of a burst release. In contrast, SET-M33D showed a slower release, with only about 50% released after 24h, suggesting substantial retention in the BC matrix.

To elucidate the mechanism underlying AMP release from BC, the obtained experimental release data were fitted to various and widely used drug release models, including zero-order, first-order, Higuchi, and KP models [62]. Mathematical model fitting revealed that the AMP release profiles from BC were best fitted to the KP, as indicated by the highest R^2^ values (Table 5). Drug release from carrier matrices can occur through several mechanisms, such as solute diffusion, matrix swelling and material degradation, depending on the nature of the material. Based on the KP model fitting, the AMP release from BC appears to be primarily diffusion-driven. In this mathematical model, the *n* value characterizes the underlying release mechanism. The *n* ≤ 0.45 indicates Fickian diffusion, while *n* between 0.45 and 0.89 corresponds to non-Fickian diffusion and values ≥ 0.89 represent case II transport or super case II transport [63]. In fact, the extremely low *n* value for both AMPs suggests that the rate of release of AMPs into the surrounding media is significantly governed by surface phenomena [64]. Furthermore, the maximum concentration in the released media was reached rapidly, implying that the diffusion path for the solute is relatively short, supporting the hypothesis that the release is primarily driven by surface interactions (potentially due to entrapment within surface porosity) rather than bulk diffusion through the BC matrix.

**Table 5:** The goodness of fit and model parameters for mathematical drug release kinetics.

Model	Zero-order		First-order		Higuchi		Korsmeyer-Peppas		
Peptide	K_0_	R^2^	K_1_	R^2^	K_H_	R^2^	K_KP_	n	R^2^
**M33-D**	0.0293	0.4822	0.0946	0.6634	0.1484	0.7252	0.4704	0.0329	0.9976
**MPX**	0.055	0.424	0.8526	0.9857	0.2700	0.6896	0.8651	0.0049	0.999

### 3.6 Combining SET-M33D with biomaterials does not induce cytotoxicity or hemolysis

Although both SET-M33D and BC have previously been reported to be biocompatible [65,66], it is important to ensure that the combination of SET-M33D and biomaterial does not raise further concerns. In fact, implanting any material into the human body leads to an immediate inflammatory response, beginning with a cascade of cellular events known as the foreign body reaction (FBR). Monocytes are among the first cells to infiltrate the implant site in response to chemokines. Here, these monocytes will differentiate into macrophages, which are essential for regulating the inflammatory response and phagocytosing pathogens [67,68]. Given their role in the successful implantation process, we determined the in vitro cytocompatibility of THP-1 monocytes with the SET-M33D-treated Ti and BC using the resazurin assay. The results showed approximately 90% THP-1 viability after a 24 h incubation (Suppl Fig. 5b). Likewise, in line with the ISO 10993-5 principles of biomaterial safety for medical devices, no adverse effect on the viability of the primary fibroblasts in direct contact with BC/SET-M33 were observed (Suppl Fig. 5c). As the level of cell viability equaled those of the control conditions, combining the peptide with the biomaterials did not cause any cytotoxic consequences. Furthermore, hemocompatibility is a key criterion, given that the biomaterial will inevitably come into contact with blood [69]. Therefore, to ensure that the newly developed AMP delivery system has no adverse effects on blood components, the interaction between SET-M33D-loaded BC and blood was evaluated at 2 × and 4 × MIC concentration of the AMP. All tested conditions showed hemolysis below 1% (Suppl Fig. 5e and f).

### 3.7 Combining BC with SET-M33D inhibits upregulation of gene involved in biofilm matrix production

To gain deeper insight into the impact of SET-M33D on the biofilm formation process on biomaterials, we analyzed the expression levels of the *Eno* and *icaA* genes. These genes are key regulators of *S. aureus* biofilm development: *Eno* encodes a protein that is responsible for adhesion to host and abiotic surfaces, while *icaA* is involved in the production of polysaccharide intercellular adhesin, a key component of the biofilm matrix. The selection of these genes was based on the observed effects of SET-M33D, as demonstrated by enumerating viable bacteria recovered from biomaterial's surfaces and by SEM. Analysis of these genes allowed us to examine how SET-M33D influences both the initial adhesion and matrix formation stages of biofilm development.

**Fig. 6:**
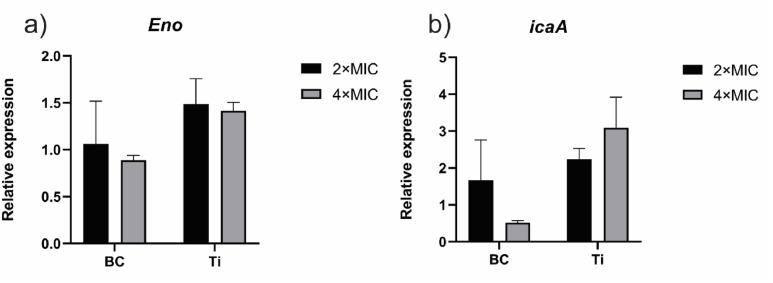
**Expression of *S. aureus* biofilm-associated genes is impacted by loading BC but not Ti with SET-M33D.** Biofilm associated genes a) *Eno*, encoding a protein involved in adhesion of *S. aureus* to host surfaces, and b) *icaA*, crucial for synthesizing polysaccharide intercellular adhesins, were assessed using RT-PCR. Data were normalized against 16S rRNA and expressed as fold change relative to *S. aureus* on untreated biomaterials. Data are expressed as the mean ± SEM of two independent experiment in quadruplicate. Statistical analysis was performed using two-way ANOVA with Sidak multiple comparison.

Gene expression analysis revealed differences in the response of *S. aureus* to SET-M33D-treated BC and Ti (Fig. 6). Treatment with 2 × MIC and 4 × MIC did not have any significant effect on the expression of *Eno* mRNA, as it remained relatively stable on BC/SET-M33D (close to untreated control). In Ti/SET-M33D condition, *Eno* expression was moderately upregulated, reaching approximately 1.5-fold relative to untreated control. Conversely, the expression of *icaA* was notably downregulated on BC at 4 × MIC, suggesting a strong inhibition of polysaccharide matrix production. In contrast, *icaA* expression remained upregulated on Ti, indicating that the biofilm forming capability of *S. aureus* persisted despite AMP treatment.

## 4 Discussion

The current study demonstrates potent activity of BC, loaded with AMPs, in particular SET-M33D, in preventing *S. aureus* biofilm formation and indicates its promising role in improving the protection of implantable medical devices against microbial colonization and subsequent infection.

Our data show that SET-M33D is more effective at inhibiting biofilm formation than MPX, particularly when used in combination with BC. This may be due to the loading efficiency and release kinetics of SET-M33D from BC. The porous, hydrophilic nature of BC allows exceptional fluid retention capacity [70]. However, this hydrophilicity could be a double-edged sword as it confers antifouling properties to the BC surface [37,71], but also allows it to absorb nutrients required for bacterial growth. Surface topography and roughness also have considerable impacts on initial bacteria adhesion, as *S. aureus* has relatively low mobility and tends to adhere and grow on surfaces with valleys and grooves [72,73]. While non-porous implantable materials such as Ti are made moderately smooth to meet antifouling characteristics, we previously demonstrated that bio-manufactured BC features a surface porosity of 11.98% and fiber diameter of 45 ± 0.013 nm [74]. Therefore, bacteria may be able to embed more effectively into the untreated BC surface and withstand hydrodynamic forces [75,76]. However, we observed that incorporating a higher concentration of AMPs, particularly SET-M33D at 4 × MIC completely inhibited bacteria colonization on the BC surface. In fact, surface charge is another well-known influential parameter that impacts bacterial attachment and biofilm formation [77]. Due to an isoelectric point <2.8, BC typically possesses a negative charge, which repels negatively charged bacteria through electrostatic repulsion [78]. However, loading a higher concentration of SET-M33D which has a strong net positive charge of +6, can alter the charge density of BC. Together with nutrients availability, the AMP-loaded BC attracts bacteria and facilitates the binding of peptide molecules to bacterial cell wall. Additionally, the porous structure of BC supports diffusion-based release of the antimicrobial agent, which is sufficient to kill planktonic bacteria in the early stages. This combined mechanism effectively prevents bacterial colonization and biofilm formation.

The inhibitory and bactericidal efficacy of AMPs is highly influenced by various factors, including length, charge and hydrophobicity which govern their interaction with bacteria [79]. While both MPX and SET-M33D are cationic (i.e. they carry a net positive charge, which is crucial for antimicrobial activity through interaction with and disruption of the negatively charged bacterial cell membrane), MPX is a simple linear peptide, composed of 14 amino acids [80]. In contrast, SET-M33D is a shorter, nine amino acid peptide, but it is tetra-branched. Hence, the higher activity of SET-M33D may be due to its architecture leading to increased and simultaneous interaction with multiple sites on the bacteria membrane, thereby enhancing its ability to compromise the membrane integrity. Additionally, the tetra-branched format of SET-M33D results in an increased and localized concentration of active sequences in a smaller area, compared to linear AMPs. Furthermore the AMP should be resistant to bacterial proteases. *S. aureus* secretes a number of proteases, including cysteine, serine and aureolysin proteases [[81], [82], [83]], which have been shown in-vitro to degrade and inactivate the linear AMP LL-37 [84]. To address this issue, SET-M33 was modified by incorporating unnatural d-amino acids [35], which enable steric hindrance to be created and binding to be minimized, as well as catalyzing the cleavage of peptide bonds [34]. The evaluation of biofilm inhibition and dynamics further highlights the importance of AMP stability and resistance to inactivation. The re-emergence of biofilm at lower active AMP concentrations after 24h, despite seemingly near-complete inhibition at 8h, indicates that surviving bacteria adapt, grow out and produce a strong biofilm. The finding that the SET-M33D peptide maintained strong biofilm inhibitory activity against *S. aureus* after 24h of incubation, demonstrates its high stability. This stability is crucial for clinical use, as it ensures sustained antimicrobial activity by maintaining an effective concentration over time, potentially reducing the need for frequent dosing.

The SEM examination of the BC surface revealed that *S. aureus* remained attached to the BC/MPX, despite the observed reduction in CFU when viable bacteria were enumerated. This raises the possibility that the discrepancy between microscopic visualization and CFU counting could be due to the bacteria entering a viable but non-culturable (VBNC) state. In this state, the bacteria are alive but do not grow on standard culture media. The VBNC state is typically induced by stressful conditions, including exposure to antimicrobial agents, and is characterized by reduced metabolic activity, limited nutrient transport, and the formation of protective structures [85]. The role of AMP treatment in inducing the VBNC state is not well understood, but a study by Vorobii et al. [86] demonstrated that 67% of *S. aureus* cells within biofilms that developed on polymer brushes immobilized with the synthetic peptide Pep19–2.5 entered the VBNC state, when compared to untreated surfaces. Alternatively, the observed discrepancy may may be due to an insufficient MPX concentration, either loaded and released from BC or bonded to the bacterial membrane. A subtherapeutic AMP concentration may fail to completely inhibit *S. aureus* adhesion or kill all bacteria upon direct contact. This can pose significant clinical challenges, including false-negative results in culture-based diagnostic methods, and recurrent infection which further complicates treatment [87]. To assess whether insufficient loading or incomplete release of AMPs contributed to the differences in antibiofilm efficacy between BC/SET-M33D and BC/MPX, we performed in vitro AMP loading and release tests. The results revealed that the amount of SET-M33D loaded into BC was approximately twice that of MPX. Furthermore, SET-M33D exhibited a slower, more sustained release profile, with approximately 50% of the loaded AMP released over 24h. In fact, observed initial burst release facilitates rapid antimicrobial action which is critical in early stages of biofilm formation through inhibiting initial adhesion and bactericidal activity. Whereas, sustained release maintains the antimicrobial level over required period to ensure long-term surface protection via preventing bacteria regrowth. These findings align with our recent study, which reported a diffusion rate of 0.7 × 10^−3^ ± 0.59 × 10^−4^ mg/h and over 50% drug retention when evaluating TAMRA-SET-M33 diffusion across the BC layer [74]. Subsequently, mathematical modeling of AMP release was conducted to identify the underlying mechanisms involved in AMP release from porous BC layer. For non-degradable biopolymers like BC, the drug release mechanism is mainly diffusion driven, with the concentration gradient playing a major role [88]. Our modeling results confirmed this, with the KP model providing the best fitting model for the experimental data. However, the obtained release constant and exponent values suggest that the majority of AMPs were entrapped within the surface porosity of BC matrix, reinforcing the role of the matrix structure in controlling release kinetics.

In contrast, increasing SET-M33D and MPX concentration up to 4 × MIC on Ti surfaces, did not lead to a substantial reduction in the number of viable bacteria. This outcome can be attributed to the intrinsic differences between BC and Ti. The porous structure of BC allows the diffusion and release of AMP into the medium, whereas Ti does not permit this. Consequently, the antimicrobial effect on Ti relies solely on the surface-immobilized AMPs, which may be insufficient to inhibit bacteria adhesion or disrupt subsequent matrix production.

To understand the limited efficacy of SET-M33D on Ti, we evaluated the expression of genes involved in adhesion and biofilm matrix. Upregulation of biofilm-associated genes on AMP-treated Ti supports the hypothesis that this treatment fails to inhibit biofilm initiation. Specifically, *Eno,* which encodes enolase, responsible enzyme for laminin-binding and adhesion, essential in the acetate pathway by converting 2-phospho-d-glycerate to phosphoenolpyruvate, which regulates the production of pyruvate and acetyl-coA [89,90] and *icaA*, part of the *icaADBC* operon responsible for synthesizing PIA, a major constituent of the EPS of *S. aureus* biofilm [91], were both upregulated. These findings reinforce the limitations of AMP treatment on non-porous metallic surfaces like Ti, even at elevated concentrations.

Our findings are consistent with those of Kuik et al., who demonstrated that initial biofilm formation by *S. aureus* on Ti surface is largely influenced by surface topology and is associated with significant upregulation of proteins involved in adhesion and metabolism [92]. As AMP-treated Ti surfaces remains vulnerable to bacteria, alternative surface modification approaches may be required. Techniques such as ion implantation, physical and chemical vapor deposition, electrophoretic deposition for coating with nitride and silver nanoparticles have been proposed to enhance the antimicrobial properties of Ti [93,94]. Although surface modification techniques can enhance the antibiofilm activity of Ti, they are often expensive, require specialized equipment, environmentally hazardous and technically challenging [95], highlighting the significance of exploiting antimicrobial-loaded envelopes in protecting non-porous implants (e.g. metals) from bacteria attachment and subsequent infection.

Altogether, these challenges highlight the potential value of antimicrobial-loaded biomaterial coatings, such as AMP-loaded BC, in protecting non-porous implants from microbial colonization. The successful integration of SET-M33D into BC and its demonstrated efficacy in preventing bacterial adhesion, underscores the promise of such systems in reducing the risk of chronic implant-associated infections. By combining controlled drug release with physical barrier properties, BC-based AMP delivery platforms offer a powerful tool to protect implants and reduce the incidence of chronic infections, initiated by biofilm development on implants.

### 4.1 Limitations

This study has several potential limitations. While it has previously been shown that the antimicrobial function of the SET-M33 was not affected by the surrounding media, particularly in protein-rich serum [96], the release of the peptide from BC in such media could be impacted by the protein concentration around the peptide-loaded BC. If occurred, this can lower the availability of the effective and active concentration of the antimicrobial peptide to interact with the target bacteria. In this work, we evaluated the release profile of the SET-M33 from BC in PBS (the standard and established method used for in vitro release studies [97]) and fitted the experimental data to the widely used mathematical models. This enabled us to determine the underlying mechanisms of peptide release from BC as a drug carrier. However, performing the same assay in a different release media, such as protein-rich serum or plasma, as well as conducting direct structural and morphological analysis (e.g. cross-sectional SEM or AFM), could further reinforce our findings regarding the loading/release and elucidate the underlying mechanism of peptide release from BC. Importantly, the activity of the antimicrobial peptides SET-M33 and MPX is unaffected by serum factors [23,96], indicating that the antimicrobial activity of these peptides is independent of the release media. Despite being improbable, another methodological constraint may pertain to the process of drying BC. Drying BC at high temperatures (typically ≥100 °C [98]) may result in a denser and less porous structure, attributable to the collapse of 3D fibrous networks. This phenomenon can result in a reduction of the loading capacity of liquid-based antimicrobials, including large molecules such as antimicrobial peptides. In the current work, we oven-dried the BC in accordance with previous reports [44,99]. This approach which operates at temperature not exceeding 80 °C, has been shown to promote efficient evaporation in a relatively short timeframe. It is acknowledged that, despite the potential for minor alterations to the microstructure of BC, resulting in increased brittleness [98], the system's inherent antimicrobial properties were preserved, as evidenced by the prevention of bacterial adhesion. This preservation of antimicrobial activity can be attributed to the high liquid uptake and retention capacity of BC. For a thorough evaluation of any alterations in material's properties that may be introduced by the process of drying at elevated temperatures, additional structural and physicochemical characterization (e.g. FTIR, XRD and SEM) of the BC is suggested.

## 5 Conclusion

Device-related infections are a major challenge that not only affects the function and lifespan of CIEDs but are also associated with increased morbidity and mortality in implant-receiving patients. *S. aureus* remains the predominant pathogen, primarily due to its ability to adhere to device surfaces and form biofilms. While porous antibiotic-eluting envelopes have been at the forefront of protecting CIEDs from infection in high risk patients, they fall short in addressing the growing concern of antibiotic resistance, highlighting the urgency of developing alternative approaches.

In this study, we investigated the potential of a synthetic AMP (SET-M33D) in combination with BC to inhibit *S. aureus* adhesion and biofilm formation, compared to non-porous Ti. Our findings are in line with other studies, and highlight the importance of biomaterial selection in ensuring long-term implant success. The porous structure of BC was shown to facilitate the diffusion of AMP, which further enabled the antimicrobial activity against *S. aureus*. In contrast, the efficacy of SET-M33D on Ti may have been limited due to the molecules bound to the surface, with limited availability to the bacterial cells.

**Fig. 7:**
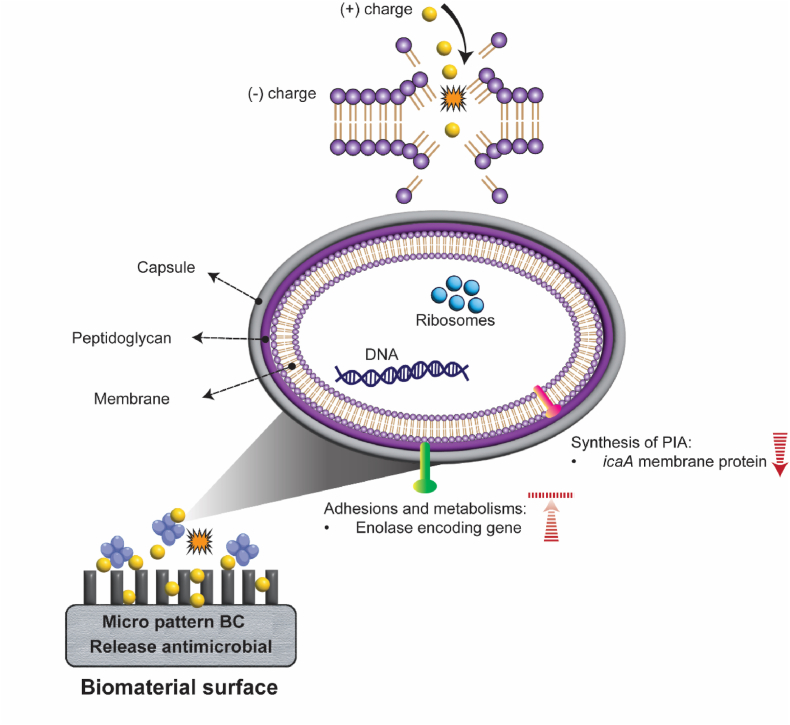
Proposed Mechanism of action of the proposed antimicrobial peptide delivery system.

At 4 × MIC, complete inhibition of bacterial adhesion (*S. aureus* ATCC29213) was observed only with the BC/SET-M33D combination. In contrast, BC/MPX still exhibited attached bacteria as shown in SEM images. SET-M33D demonstrated superior performance likely due to its protease-resistant, multivalent structure and its interaction with bacterial membranes—particularly with components like lipoteichoic acid. (Fig. 7). As demonstrated here, the antibiofilm activity of SET-M33D/BC may be exerted through the downregulation of genes involved in adhesion, metabolism, and EPS (e.g. polysaccharide intercellular adhesin) synthesis. Further assessments such as whole cell transcriptomics, could provide a more comprehensive understanding of SET-M33D/BC exposure on gene expression, and specifically biofilm-associated genes of *S. aureus*. Mu50 and MW2 MRSA clinical isolates In particular, have been shown to be less vulnerable to the SET-M33/BC combination than standard strain ATCC29213. This could be due to their genetic profile. It would therefore be worthwhile to explore whether loading BC with SET-M33 to protect CIEDs, could lead to the downregulation or prevention of the upregulation of biofilm-associated genes in these MRSA strains.

Despite its effectiveness, the drug release studies revealed that BC retained a large proportion of SET-M33D, likely due to its large tetra-branched structure as well as the tortuous assembly of BC. This limited its full antimicrobial potential, especially at lower concentrations. Therefore, further optimization of the BC matrix, the AMP structure, or both is necessary to achieve sustained release of AMP at therapeutic levels at the implant site for the required duration.

## CRediT authorship contribution statement

**Sajad Mohammadi:** Writing – review & editing, Writing – original draft, Visualization, Validation, Methodology, Data curation, Conceptualization. **Alessia Maranesi:** Methodology, Data curation, Conceptualization. **Adrianus C.J.M. de Bruijn:** Methodology. **Ismael Castañon:** Methodology. **Piotr Gierlich:** Methodology. **Chiara Falciani:** Methodology. **Alessandro Pini:** Methodology. **Heleen M.M. van Beusekom:** Methodology. **Aldo Ferrari:** Writing – review & editing, Validation, Supervision, Resources, Methodology, Conceptualization. **Wendy W.J. Unger:** Writing – review & editing, Validation, Supervision, Resources, Project administration, Methodology, Funding acquisition, Conceptualization.

## Funding

This work was supported by European Union Horizon Europe MSCA programme (grant agreement number 101073263 [SSBB]).

## Data Availability

Data will be made available on request.
