# Supplementary material for: SET-M33 loaded biosynthesized cellulose as effective protection against S. aureus biofilm formation
Source: Biofilm. 2026 Feb 2;11:100351. doi: 10.1016/j.bioflm.2026.100351 (PMC12914680; doi:10.1016/j.bioflm.2026.100351)
Supplement: Multimedia component 1 [file mmc1.docx]

**SET-M33 loaded biosynthesized cellulose as effective protection against *S. aureus* biofilm formation**


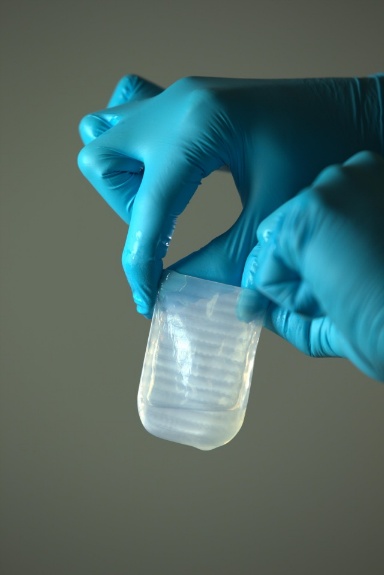


**Suppl Figure 1.** Microstructured biocellulose envelope produced via guided-assembly biolithography (GAB)

**Minimum Inhibitory and bactericidal Concentration of SET-M33D and MPX**

The Minimum Inhibitory Concentration (MIC) of the antimicrobial peptide SET-M33D and MPX against *Staphylococcus aureus* ATCC29213, MRSA (MW2 and Mu50) and MSSA (LVAD) clinical isolates was determined using a broth microdilution method, in accordance with the guidelines recommended by EUCAST (<https://www.eucast.org/>), with some modifications. Briefly, *S. aureus* cultures were prepared in LB broth at a concentration of 1.5×10^6^ CFU/mL, and a two-fold serial dilution of the SET-M33D and MPX peptides was performed to achieve a concentration range from 42.68 to 0.041 and 80.33 to 0.062 µM, respectively. Each well in a flat-bottom 96-well microtiter plate was inoculated with 100 µL of the bacterial suspension and 100 µL of the peptide solution, resulting in a final volume of 200 µL per well. Controls included LB alone, LB with bacteria, and LB with bacteria plus rifampin. Plates were incubated at 37°C for 18-24 h, after which the optical density at 600 nm was determined using a microplate reader (VersaMax, Molecular Devices). The MIC was defined as the lowest concentration of peptide that inhibited growth of *S. aureus*.

The minimum bactericidal concentrations (MBC) were determined by subculture of 50 µL of samples at and above the MIC onto antibiotic-free agar plate. The agar plates were incubated at 37°C for 24 h, after which the viable CFU/mL was enumerated. The lowest concentrations that demonstrated 99.9% reduction of the final inoculum were considered as MBC values.

**Suppl Table 1**. **Minimum inhibitory concentration (MIC) and minimum bactericidal concentration (MBC) of SET-M33D, MPX and Rifampicin against *S. aureus* strains, including clinical isolates used in this study**

| *S. aureus* strains | SET-M33D (µM) | | MPX(µM) | | Rifampicin(µM) | |
| --- | --- | --- | --- | --- | --- | --- |
|  | MIC | MBC | MIC | MBC | MIC | MBC |
| ATCC29213 | 2.66 | 5.32 | 16 | 32 | 0.0047 | 0.01899 |
| Mu50 | 10.67 | 42.68 | 40.17 | 80.33 | Resistant | Resistant |
| MW2 | 10.67 | 10.67 | 20.08 | 80.33 | 0.038 | 0.038 |
| LVAD | 10.67 | 10.67 | NA | NA | 0.038 | 0.038 |


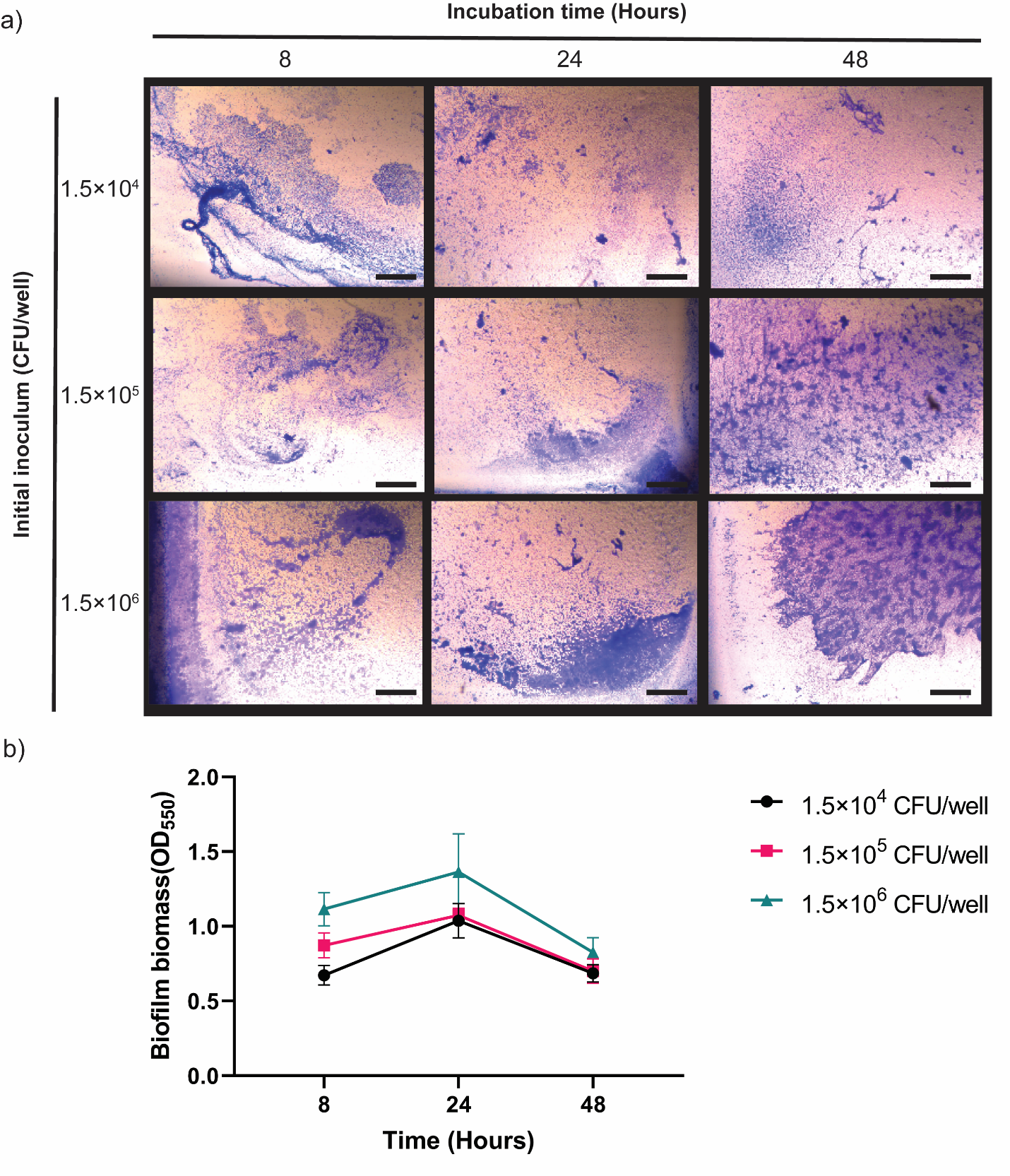


**Suppl Figure 2.** **Impact of initial load and dynamics of biofilm formation:** a) Representative microscopic images of established biofilm, stained with crystal violet. Scale bar 500 µm. b) Quantification of the biofilm biomass at 8 h, 24 h and 48 h. Mean ± SEM of two independent experiment each in duplicate.

**Suppl Table 2. Experimental runs of full factorial design of experiment**

| **Run** | **Factor A:**  **Biomaterial** | **Factor B:**  **Antimicrobial peptide** | **Factor C:**  **Concentration (×MIC**) |
| --- | --- | --- | --- |
| 1 | Ti | SET-M33D | 1 |
| 2 | Ti | MPX | 1 |
| 3 | BC | SET-M33D | 1 |
| 4 | BC | MPX | 4 |
| 5 | BC | SET-M33D | 4 |
| 6 | BC | MPX | 2 |
| 7 | BC | MPX | 2 |
| 8 | BC | SET-M33D | 2 |
| 9 | Ti | SET-M33D | 2 |
| 10 | Ti | SET-M33D | 2 |
| 11 | Ti | MPX | 2 |
| 12 | Ti | MPX | 1 |
| 13 | Ti | SET-M33D | 1 |
| 14 | Ti | MPX | 2 |
| 15 | BC | MPX | 1 |
| 16 | Ti | MPX | 4 |
| 17 | BC | SET-M33D | 2 |
| 18 | Ti | MPX | 4 |
| 19 | BC | MPX | 4 |
| 20 | Ti | SET-M33D | 4 |
| 21 | BC | SET-M33D | 1 |
| 22 | Ti | SET-M33D | 4 |
| 23 | BC | SET-M33D | 4 |
| 24 | BC | MPX | 1 |





**Suppl Figure 3. SEM visualization of inhibition of bacterial adhesion and biofilm formation on BC treated with 1µM rifampin as control, indicating similar performance compared to SET-M33D-loaded BC at 4×MIC.**


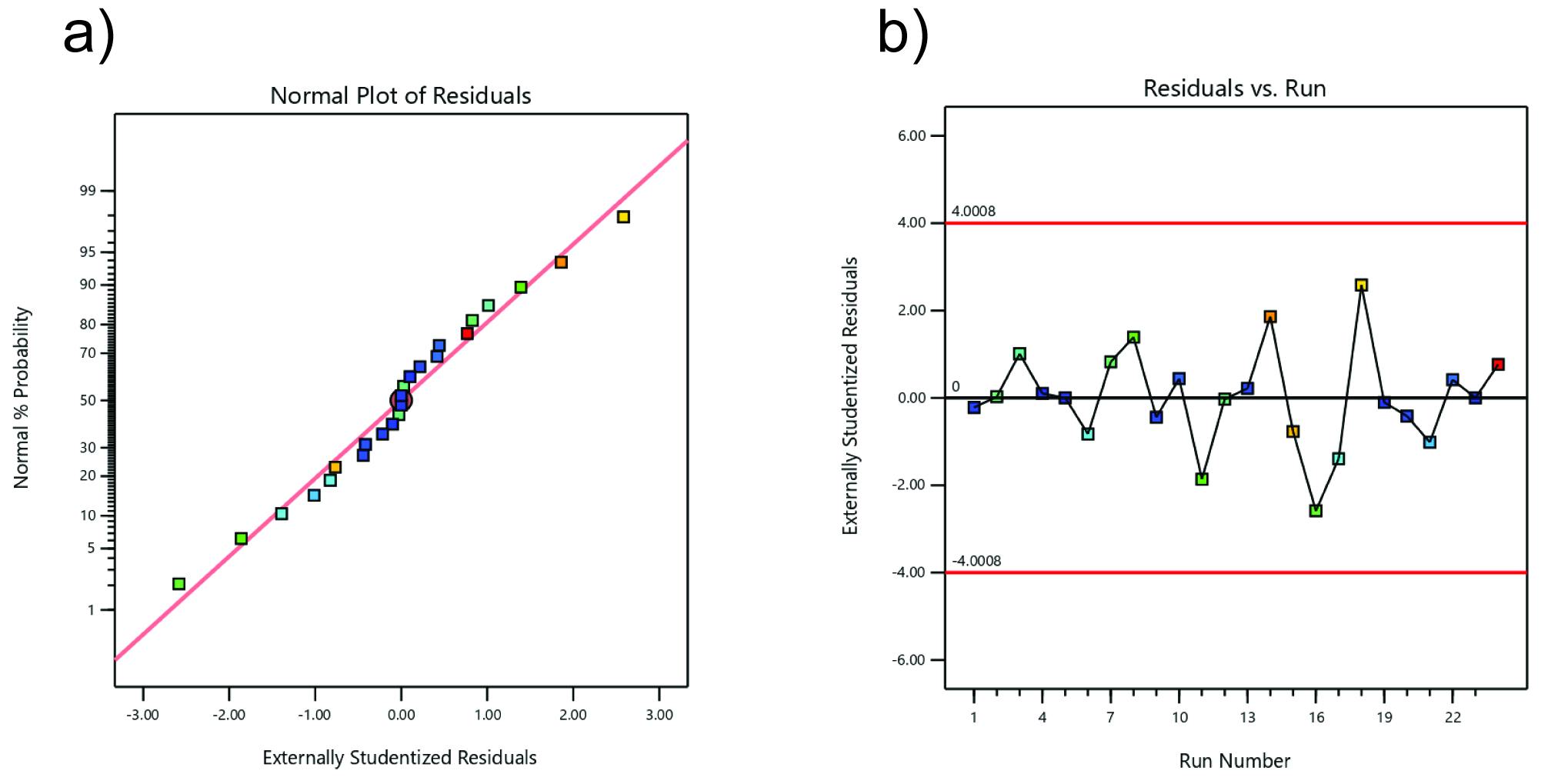


**Suppl Figure 4. Normality and residuals assessment of the statistical model:** a) The normal probability plot indicates the residuals follow a normal distribution. b) Plot of the residuals vs experimental run.

**Suppl Table3. Statistical fit table demonstrating the goodness of fit for linear regression model**

| **Std. Dev.** | **Mean** | **C.V%** | **R^2^** | **Adjusted R^2^** | **Predicted R²** | **Adeq Precision** |
| --- | --- | --- | --- | --- | --- | --- |
| 123.35 | 644.10 | 19.15 | 0.9546 | 0.9131 | 0.8186 | 15.147 |

**Combining SET-M33D with biomaterials does not induce cytotoxicity or hemolysis**


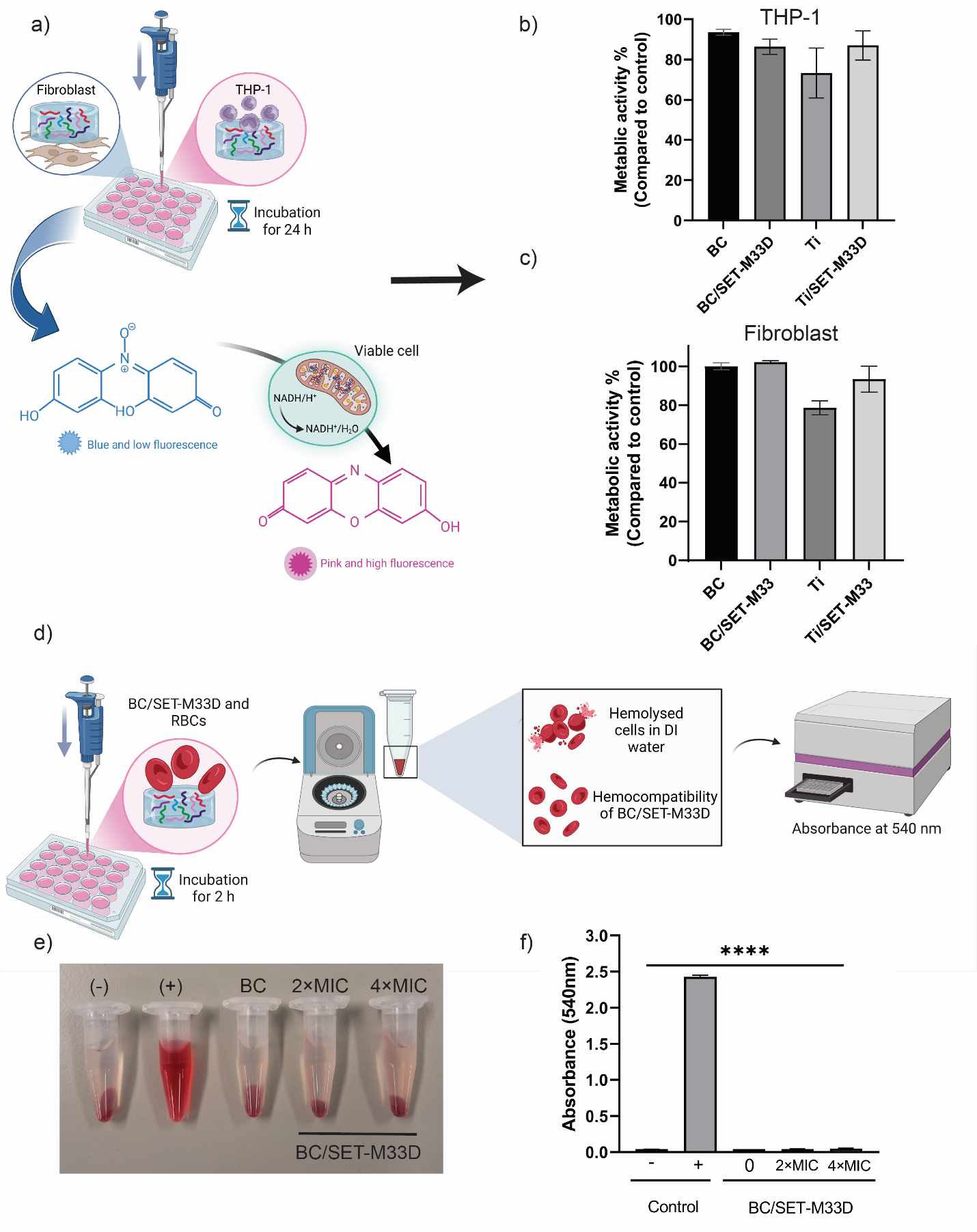


**Suppl Figure 5. Biocompatibility assessment following the incorporation of SET-M33D into biomaterials.** a) Co-culturing THP-1 and fibroblast cells with 15 µM SET-M33-loaded/treated biomaterial. b) Metabolic activity of THP-1 cells after 24 h of incubation with AMP-treated biomaterial where combination of SET-M33 with biomaterials did not induce any adverse effect. c) Metabolic activity of fibroblasts after 24 h of incubation with AMP-treated biomaterial. Hemocompatibility of the BC/SET-M33D. d) Experimental schematic of hemocompatibility test. e) Release of hemoglobin from hemolyzed cells into supernatant. f) Absorbance of supernatant indicating equal amount of hemoglobin release for BC/SET-M33 and negative control. Mean ± SEM. (****) p<0.0001.
